# Correction: Ngo, T.D. LinkMind: Link Optimization in Swarming Mobile Sensor Networks. *Sensors* 2011, *11*, 8180–8202

**DOI:** 10.3390/s111110165

**Published:** 2011-10-26

**Authors:** Trung Dung Ngo

**Affiliations:** 1 The More-Than-One Robotics lab, Department of Electronic Systems, Automation and Control Section, Aalborg University, Fredrik Bajers Vej 7C, 9220 Aalborg, Denmark; 2 Faculty of Science, University of Brunei Darussalam, Jalan Tungku Link, Gadong, BE1410, Brunei

The author’s affiliation was wrong in [1]. The “National University of Brunei” and should be “University of Brunei Darussalam”.
